# Environment and Colonisation Sequence Are Key Parameters Driving Cooperation and Competition between *Pseudomonas aeruginosa* Cystic Fibrosis Strains and Oral Commensal Streptococci

**DOI:** 10.1371/journal.pone.0115513

**Published:** 2015-02-24

**Authors:** Robert A. Whiley, Emily V. Fleming, Ridhima Makhija, Richard D. Waite

**Affiliations:** 1 Department of Clinical & Diagnostic Oral Sciences, Barts and The London School of Medicine and Dentistry, Queen Mary University of London, 4 Newark Street, London, United Kingdom, E1 2AT; 2 Centre for Immunology and Infectious Disease, Blizard Institute, Barts and The London School of Medicine and Dentistry, Queen Mary University of London, 4 Newark Street, London, United Kingdom, E1 2AT; University Hospital of the Albert-Ludwigs-University Freiburg, GERMANY

## Abstract

Cystic fibrosis (CF) patient airways harbour diverse microbial consortia that, in addition to the recognized principal pathogen *Pseudomonas aeruginosa*, include other bacteria commonly regarded as commensals. The latter include the oral (viridans) streptococci, which recent evidence indicates play an active role during infection of this environmentally diverse niche. As the interactions between inhabitants of the CF airway can potentially alter disease progression, it is important to identify key cooperators/competitors and environmental influences if therapeutic intervention is to be improved and pulmonary decline arrested. Importantly, we recently showed that virulence of the *P*. *aeruginosa* Liverpool Epidemic Strain (LES) could be potentiated by the Anginosus-group of streptococci (AGS). In the present study we explored the relationships between other viridans streptococci (*Streptococcus oralis*, *Streptococcus mitis*, *Streptococcus gordonii* and *Streptococcus sanguinis*) and the LES and observed that co-culture outcome was dependent upon inoculation sequence and environment. All four streptococcal species were shown to potentiate LES virulence factor production in co-culture biofilms. However, in the case of *S*. *oralis* interactions were environmentally determined; in air cooperation within a high cell density co-culture biofilm occurred together with stimulation of LES virulence factor production, while in an atmosphere containing added CO_2_ this species became a competitor antagonising LES growth through hydrogen peroxide (H_2_O_2_) production, significantly altering biofilm population dynamics and appearance. *Streptococcus mitis*, *S*. *gordonii* and *S*. *sanguinis* were also capable of H2O2 mediated inhibition of *P*. *aeruginosa* growth, but this was only visible when inoculated as a primary coloniser prior to introduction of the LES. Therefore, these observations, which are made in conditions relevant to the biology of CF disease pathogenesis, show that the pathogenic and colonisation potential of *P*. *aeruginosa* isolates can be modulated positively and negatively by the presence of oral commensal streptococci.

## Introduction

The airways of cystic fibrosis (CF) patients harbour a diverse consortium of microorganisms associated with disease pathogenesis and decline in lung function. However, despite the wealth of information documenting recognised CF pathogens and a more recent awareness of a potential role for other CF associated bacteria in disease progression the interactions between community members of this lung microbiome and how they are environmentally influenced remain largely unknown. Understanding the key interactors and the relationships that drive the population dynamics within these complex microbial communities will prove important in identification of inhabitants associated with both clinical stability and pulmonary exacerbation, and thus the development of improved, targeted CF therapies [1,2].

Streptococci have been frequently isolated during deep sequencing, culture-dependent and culture-independent studies on CF and non-CF respiratory microbiome composition and the role of the Anginosus-group of oral commensal streptococci (also known as the *Streptococcus milleri* group) has recently been highlighted [3,4,5,6,7,8,9]. Importantly *in-vivo* longitudinal studies have observed the proliferation of AGS to a significant proportion of the total bacterial flora before onset and during acute exacerbation in CF patients, who responded best to antibiotic therapy targeting AGS rather than *Pseudomonas aeruginosa* the established principal pathogen in adult CF [6]. Unfortunately, these commensal streptococci are still regarded as oral contaminants during routine clinical screening of CF sputa, thus their contribution to disease progression is underestimated and a potentially important therapeutic target is often ignored.

Recently, it was discovered that virulence of *P*. *aeruginosa* strain PAO1 (originally a wound isolate) in infection models could be modulated by streptococci and other oropharyngeal flora (OF) [10,11]. Due to the enormous variation in the pathogenic potential and phenotypes of *P*. *aeruginosa* isolates from different habitats and clinical origins we expanded these studies to investigate the interactions between AGS and clinical *P*. *aeruginosa* isolates with phenotypes associated with CF airway disease (low, intermediate and over-production of the virulence factor pyocyanin) [12,13,14,15]. In addition we chose to use examples of the Liverpool epidemic strain (LES) which is a highly transmissible, CF lung adapted strain frequently isolated within the United Kingdom and now found in North America [12,16]. We showed that AGS survived at high cell densities in biofilm co-culture with *P*. *aeruginosa*, that coexistence of AGS with a low and an intermediate pyocyanin producer resulted in enhancement of virulence factor production (pyocyanin and elastase) and that LES/AGS partnerships were pathogenic *in vivo* in an insect acute infection model [15]. Given the widespread association of streptococci within CF patient lung microbiomes, the aim of this study was to examine population dynamics of other oropharyngeal commensal streptococci in combination with the LES. This study extends our investigations to another streptococcal species group that mainly consists of ubiquitous oral commensals, namely the Mitis Group. These streptococci are present in high numbers and colonise all surfaces of the mouth. They are presumed to have importance in the microbial ecology of the oral cavity and include opportunistic pathogens causing endocarditis in contrast to the AGS which are associated with intra- and extra-oral purulent infections. We show that potentiation of virulence of *P*. *aeruginosa* CF isolates is not exclusive to the AGS and that, importantly, environmentally influenced H_2_O_2_ production by *Streptococcus oralis* can profoundly alter biofilm development and climax population composition.

## Materials and Methods

### Bacterial strains and culture conditions

The bacterial strains used in this study are listed in Table 1. *P*. *aeruginosa* was routinely grown at 37°C on LB agar (Invitrogen, Paisley, United Kingdom) whilst streptococcal strains were grown on blood agar containing 6% defibrinated horse blood (BA; Oxoid, Hampshire, UK) in an anaerobic atmosphere (80% nitrogen, 10% hydrogen and 10% carbon dioxide, vol/vol). Liquid cultures used to inoculate biofilms were grown overnight at 37°C in Todd Hewitt broth (Oxoid) supplemented with 0.5% Yeast Extract (BBL, Becton Dickson & Co., USA) (THY); *P*. *aeruginosa* were incubated aerobically with agitation (200 rpm) and streptococci at 37°C anaerobically.

**Table 1:** Bacterial strains.

Strain	Relevant characteristics / clinical source	Reference
*P*. *aeruginosa*:		
CF2004	LES non-mucoid CF isolate (*lasR* mutant, low pyocyanin producer)	[15]
768	LES non-mucoid CF isolate (*lasR* mutant, pyocyanin overproducer)	[15]
H129	LES non-mucoid CF isolate (intermediate pyocyanin producer)	[22]
DWW2	Mucoid CF isolate	This study
*S*. *oralis*		
ATCC35037^T^	Type strain LVG1	
ATCC35037^T^ *spxB*	*spxB* deficient mutant, decreased production of H_2_O_2_.	[35]
*S*. *mitis*		
NCTC 12261^T^	Type strain NS51	NCTC
*S*. *gordonii*		
NCTC 7865^T^	Type strain	NCTC
*S*. *sanguinis*		
NCTC 7863^T^	Type strain	NCTC

NCTC = National Collection of Type Cultures

^T^ = Type strain

### Biofilm modelling

Static biofilms were grown on 47 mm diameter nitrocellulose filters (Millipore) placed on THY agar as previously described [15]. Replicate filters were inoculated with approximately 7.0x10^4^–1.0x10^5^ colony-forming units (CFU) of a *P*. *aeruginosa* isolate either singly or in combination with 5–24 fold more streptococcal CFU (co-cultures). Aerobic incubation was at 37°C in either a 10% CO_2_ atmosphere or in the absence of added CO_2_. After 48 h incubation, filters were placed into 10 ml THY broth, vortexed for 2x30 seconds interspersed by scraping with a sterile loop, to recover the bacteria from the biofilms. For bacterial population quantitation, viable counts were performed using Pseudomonas Isolation Agar (PIA; Difco) incubated aerobically overnight (37°C) and TYC medium (Lab M, Heywood, Lancashire, UK) incubated anaerobically (37°C) for a minimum of 24 h to select for streptococci. To assess the effect of H_2_O_2_ decomposition on population dynamics in *P*. *aeruginosa* / *S*. *oralis* co-culture biofilms 500 units of catalase (from bovine liver, Sigma Aldrich) was incorporated into the inoculum before spreading onto the nitrocellulose filters.

### Detection of *P*. *aeruginosa* virulence factor production

Virulence factor quantification was performed as described previously using supernatant generated from biofilms grown and disrupted as described above [15,17,18].

### Hydrogen peroxide (H_2_O_2_) quantification

Triplicate filters were inoculated with approximately 5x10^5^ CFU of a streptococcal strain and incubated at 37°C in either a 5% or 10% CO_2_ atmosphere or in the absence of added CO_2_. After 24 h incubation filters were placed into 2.5 ml sterile saline and bacterial cells removed as above. Resuspended biofilm cells were pelleted by centrifugation and H_2_O_2_ quantified in 50 μl aliquots of supernatant using a hydrogen peroxide colorimetric detection kit (Enzo Life Sciences Ltd., Exeter, UK.)

### Pioneer / secondary coloniser competition assays

Antagonism of growth of competitors by pioneering streptococcal colonisers was assessed using a method adapted from a previously described protocol [19]. Briefly, 8 μl of streptococcal overnight cultures were inoculated onto THY agar and incubated at 37°C h in an aerobic (+10% CO_2_) or an anaerobic atmosphere. After 24 h, 8 μl of a *P*. *aeruginosa* CF2004 overnight culture (diluted 1000 fold) was inoculated next to the streptococcal colonies formed and agar plates were incubated for a further 24 h at 37°C h in an aerobic atmosphere (+10% CO_2_). Growth antagonism was revealed by the inhibition of growth of the side of the *P*. *aeruginosa* colony proximal to the streptococcal colony.

### Statistical Analysis

Data were analysed using one-way analysis of variance (ANOVA) with Holm-Sidak post hoc test or the Student’s t-test (two independent samples assuming unequal variances). Differences were statistically significant at *p*<0.05*, p<0.01**, p<0.001***. The degree of spread of the data is shown as standard deviation.

## Results

### Population densities and virulence factor production in co-culture biofilms

Streptococcal species belonging to the Mitis species group were selected for this study as these are amongst the most common taxa found both in the oropharyngeal flora and from the sputa of CF patients [20,21]. CF2004, the *P*. *aeruginosa* strain chosen is a low virulence factor producing variant of a common subtype of the LES, and we recently showed that its virulence could be potentiated by the presence of AGS [15].

**Fig 1:**
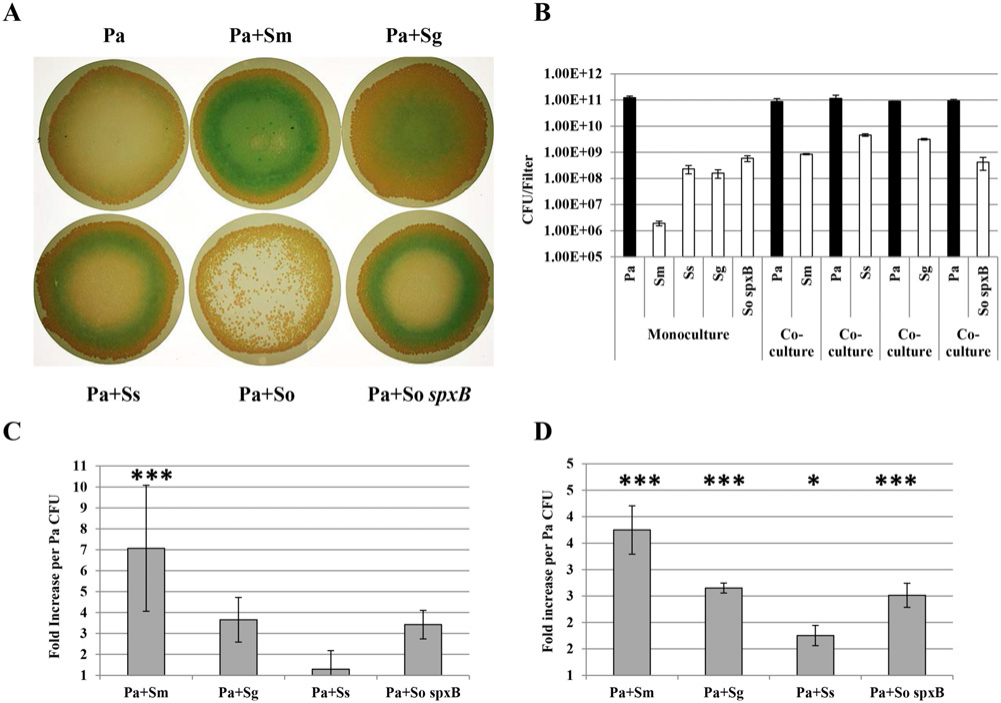
Population dynamics and virulence factor production in co-culture biofilms. Bacterial strains—Pa, *P*. *aeruginosa* CF2004; Sm, *S*. *mitis* NCTC 12261^T^; Sg, *S*. *gordonii* NCTC 7865^T^; Ss, *S*. *sanguinis* NCTC 7863^T^; So, *S*. *oralis* ATCC 35037^T^ and So *spxB*, *S*. *oralis* ATCC 35037^T^ *spxB* mutant. Inoculation ratios for Pa:Sm, Pa:Sg, Pa:Ss, Pa:So and Pa:So *spxB* were 1:6, 1:18, 1:24, 1:6 and 1:5 respectively. (A) Biofilms formed on nitrocellulose filters after 48 h (37°C, 10% CO_2_). (B) Quantitative bacteriology (CFU / filter) of monoculture and co-culture biofilms sampled after 48 h (at 37°C, 10% CO_2_); shaded bars *P*. *aeruginosa* CF2004 population, unshaded bars streptococcal population; significant differences between cfu in monocultures and co-cultures (*p*<0.001***) using two-tailed t-test were observed for all streptococci with the exception of the *S*.*oralis spxB* mutant. (C) Pyocyanin and (D) elastase activity per *P*. *aeruginosa* cfu. Fold increase in co-culture biofilms compared with mono-culture control displayed. Biofilms sampled after 48 h (at 37°C); standard deviation of triplicate or quadruplicate cultures shown. Results significantly different from control using ANOVA with Holm Sidak post hoc test are denoted with an asterisk (*p<0.05, ***p<0.001). A significant fold-increase in pyocyanin was also observed between the Pa+Sm / Pa+Ss pairing (*p*<0.001) and for elastase production significant increases were observed for the following pairings; Pa+Sm / Pa+Ss, Pa+Sm / Pa+So *spxB* (*p*<0.001), Pa+Sm / Pa+Sg (*p*<0.01) and Pa+Sg / Pa+Ss (*p*<0.05).

*P*. *aeruginosa* CF2004 was grown in monoculture and in co-culture with *Streptococcus mitis* NCTC 12261^T^, *Streptococcus gordonii* NCTC 7865^T^, *Streptococcus sanguinis* NCTC 7863^T^ and *S*. *oralis* ATCC 35037^T^ under the same environmental conditions used in our previous study -37°C, aerobically with a 10% CO_2_ atmosphere (+10% CO_2_) [15] (Fig. 1A). As for previous observations with AGS, *S*. *mitis*, *S*. *gordonii* and *S*. *sanguinis* all survived at high cell density in biofilm co-culture achieving populations of 8.5x10^8–^4.6x10^9^ CFU / filter after 48 h (20–440 fold higher than in monoculture, *p*<0.001), with CF2004 always the numerically dominant partner (8.8x10^10–^1.2x10^11^ CFU filter) (Fig. 1B). Again as with AGS, when in co-culture with *S*. *mitis*, CF2004 clearly displayed a more uniform and intense distribution of blue-green pigmentation than in monoculture biofilms, which was reflected in a significantly increased measurement of the virulence factor pyocyanin (Fig. 1A and 1C, F_(4,19)_ = 8.634; *p*<0.001). In addition, elastase production was also significantly enhanced in CF2004 / *S*. *mitis* co-culture biofilms (F_(4,14) = 26.226;_ *p*<0.001) and in combinations containing *S*. *gordonii* (p<0.001) and *S*. *sanguinis*, (p<0.05) (Fig. 1D).

**Fig 2:**
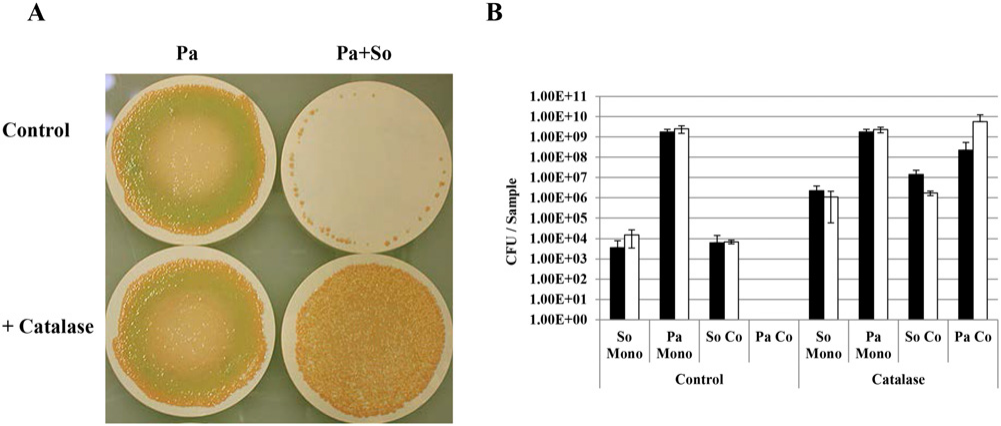
Catalase-mediated protection of the CF2004 population when grown in biofilm co-culture with *S*. *oralis*. Bacterial strains—Pa, *P*. *aeruginosa* CF2004; So, *S*. *oralis* ATCC 35037^T^. A) Biofilms grown on nitrocellulose filters for 48 h (37°C, 10% CO_2_) in the presence and absence of catalase (500 units). (B) Quantitative bacteriology (CFU / filter) of samples taken from central regions of biofilms (+ or - catalase) after 24 and 48 h at 37°C (+10% CO_2_); shaded bars 24 h population, unshaded bars 48 h population. Detection limit; 1.0x10^3^ CFU/ sample.

The co-culture biofilm that showed the most striking difference, was the CF2004 / *S*. *oralis* combination. For these biofilms *P*. *aeruginosa* colonies grew mostly around the periphery of the filter with small *S*. *oralis* colonies just visible to the naked eye covering the remaining surface (Fig. 1A). As *S*. *oralis* is a known producer of H_2_O_2_ we hypothesised that this was the antimicrobial factor responsible for the prevention of development of a confluent biofilm with *P*. *aeruginosa* as the principal component. This was confirmed when monoculture biofilm levels of *P*. *aeruginosa* population were recovered when CF2004 was grown in combination with a *S*. *oralis* mutant deficient in H_2_O_2_ production (*S*. *oralis* ATCC 35037 *spxB* deletion mutant) (Fig. 1A and 1B). Interestingly, biofilm co-culture with the *spxB* deletion mutant led to observations similar to those obtained in combination with the other wild type streptococcal species examined; high streptococcal cell density (4.2x10^8^ CFU / filter) and enhanced pyocyanin and elastase levels (*p*<0.001) (Fig. 1B, 1C and 1D). Similarly, we also observed catalase-mediated protection of the CF2004 population when grown in the presence of wild type *S*. *oralis* (Fig. 2A and 2B). In addition, *S*. *oralis* was found to survive at significantly higher cell densities in catalase protected monocultures and co-cultures (7–615 fold and 240–2220 fold higher CFU / sample, respectively) suggesting a role for H_2_O_2_ in growth limitation (Fig. 2B).

### Quantification of H_2_O_2_ levels demonstrate *S*. *oralis* ATCC 35037^T^ to be a high producer in mono-culture biofilms

**Fig 3:**
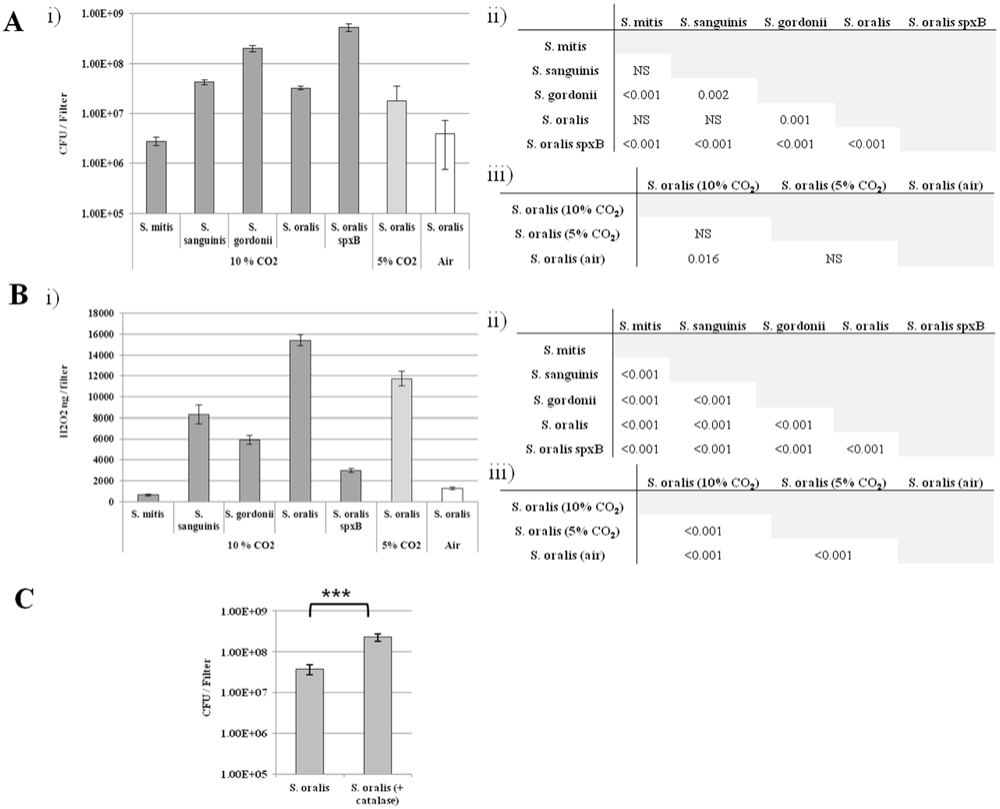
Quantitative bacteriology and H_2_O_2_ production in streptococcal mono-culture biofilms. A) Quantitative bacteriology and B) H_2_O_2_ production in monoculture biofilms sampled after 24 h incubation at 37°C in the indicated atmosphere. p values using ANOVA and Holm-Sidak post hoc test for pairwise comparisons of the quantitative bacteriology data and H_2_O_2_ production are shown in accompanying matrices (Aii, Aiii, Bii and Biii). C) Quantitative bacteriology of *Streptococcus oralis* monoculture biofilms with and without catalase. Significant difference (p<0.001***) using the two-tailed t-test shown.

Mono-culture biofilms were grown aerobically (+10% CO_2_) for 24 h for all four species of streptococci and the *S*. *oralis spxB* deletion mutant, their populations determined and the amount of H_2_O_2_ produced quantified. Variable populations were observed between species (population densities: *S*. *oralis spxB > S*. *gordonii* > *S*. *sanguinis* > *S*. *oralis* > *S*. *mitis*) with *S*. *mitis* (2.8 x 10^6^ CFU / filter) the least populated biofilm and the *S*. *oralis* ATCC 35037 *spxB* deletion mutant biofilm (5.3 x 10^8^ CFU / filter) >16 fold higher in cell density than its parent population (Fig. 3A, F_(4,10)_ = 71.11; *p*<0.001). When H_2_O_2_ was quantified, it was found to be released in monoculture biofilms of all species and despite producing the second lowest biofilm biomass *S*. *oralis* was by the far the highest producer (H_2_O_2_ production: *S*. *oralis* > *S*. *sanguinis* > *S*. *gordonii* > *S*. *oralis spxB* > *S*. *mitis*, all pairwise comparisons significantly different—F_(4,10)_ = 376.2; *p*<0.001) generating almost twice the amount of the next highest producer *S*. *sanguinis* (Fig. 3B). Unsurprisingly *S*. *oralis* was still the highest producer when H_2_O_2_ / CFU was determined and this analysis clearly showed that H_2_O_2_ is produced in the low cell density *S*. *mitis* biofilms (H_2_O_2_ / million CFU: *S*. *oralis* > *S*. *mitis* > *S*. *sanguinis* > *S*.*gordonii > S*. *oralis spxB*) (S1 Fig.).

We also evaluated biofilm cell densities and the amount of H_2_O_2_ produced by *S*. *oralis* under two other atmospheric conditions; aerobic (air) or aerobic + 5% CO_2_ (37°C) (Fig. 3A and 3B). Although the average *S*. *oralis* biomass halved in aerobic (+ 5% CO_2_) conditions the amount of H_2_O_2_ produced was still higher than that of all other streptococcal species when grown in aerobic (+ 10% CO_2_) conditions. In air however, further reductions in biofilm cell density (F_(2,6)_ = 5.434; p = 0.045) and production of H_2_O_2_ (F_(2,6)_ = 627.761; p<0.001) (>8 and >12 fold reduced, compared to + 10% CO_2_ conditions respectively) were observed.

These data explain why *S*. *oralis* is able to shape the bacterial composition of co-culture biofilms so dramatically to its advantage when grown in the presence of added CO_2_ (Fig. 1A and S1 Fig.) and show that reducing the CO_2_ concentration progressively to atmospheric levels reduces biofilm cell density and thus the amount of H_2_O_2_ produced (Fig. 3A and 3B). Interestingly, we also showed that catalase protected *S*. *oralis* biofilms had a cell density that was 6 fold higher than the parental control (Fig. 3C, *p*<0.001). This is in agreement with the higher cell density observation made with the *spxB* mutant (Fig. 3A) and in catalase protected *S*. *oralis* biofilm samples (Fig. 2B) and demonstrates that H_2_O_2_ production is a mechanism for self-limitation of *S*. *oralis* biofilm development.

### Inoculation sequence influences *P*. *aeruginosa* antagonism

**Fig 4:**
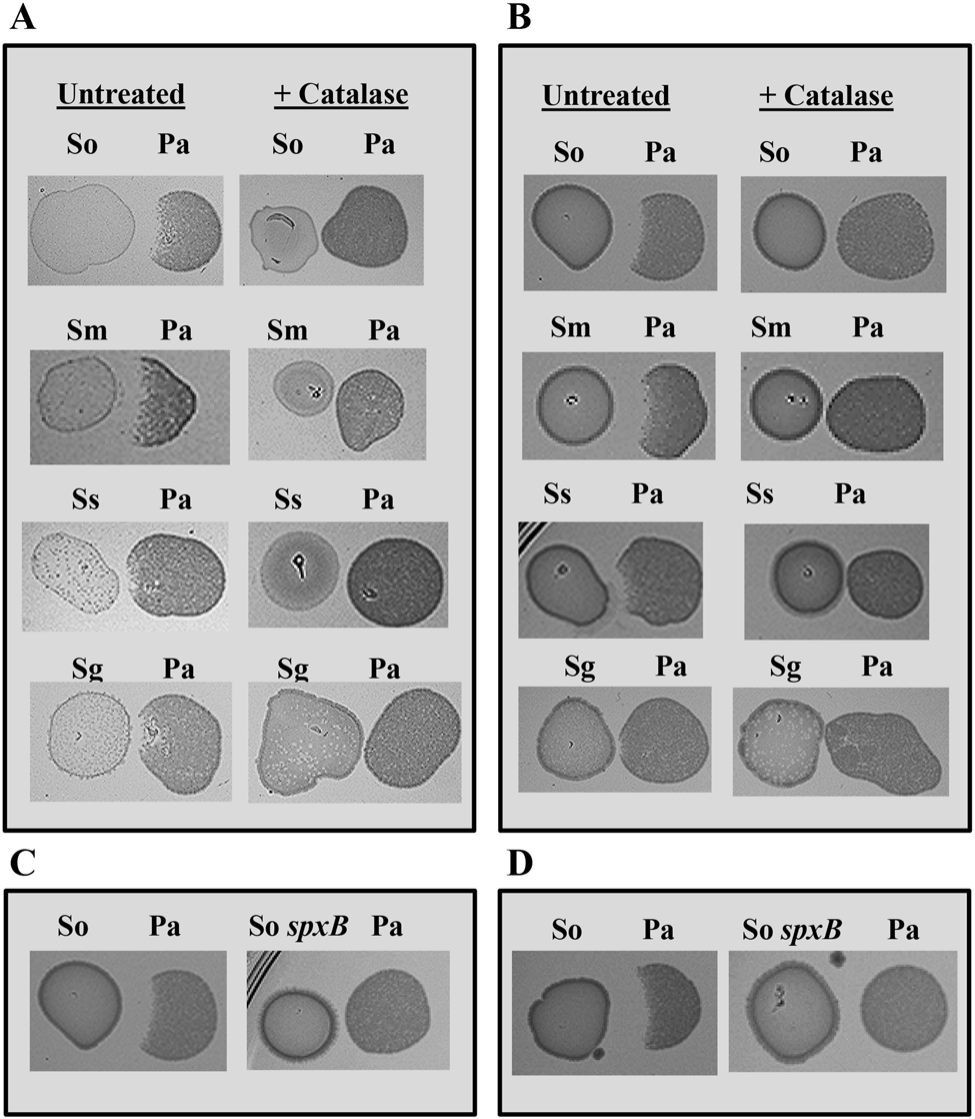
Antagonism of *P*. *aeruginosa* growth by pioneering streptococcal colonisers. Bacterial strains—Pa, *P*. *aeruginosa* CF2004; Sm, *S*. *mitis* NCTC 12261^T^; Sg, *S*. *gordonii* NCTC 7865^T^; Ss, *S*. *sanguinis* NCTC 7863^T^; So, *S*. *oralis* ATCC 35037^T^, and So *spxB*, *S*. *oralis* ATCC 35037^T^ *spxB* mutant. Streptococcal spp were inoculated onto the agar surface as pioneer colonisers and incubated for 24 h at 37°C h in an A and C) aerobic (+10% CO_2_) and B and D) anaerobic atmosphere before CF2004 cultures were adjacently inoculated as secondary colonisers and grown for a further 24 h at 37°C h in an aerobic atmosphere (+10% CO_2_).

In addition to determining population dynamics after co-inoculation in a biofilm model, we also explored whether H_2_O_2_ production by these four species of streptococci when grown as a pioneer coloniser could antagonize growth of *P*. *aeruginosa*. Streptococci were deposited on the surface of THY agar and grown for 24 h in both an aerobic (+10% CO_2_) and an anaerobic atmosphere. The compact high cellular density colonies were then challenged with *P*. *aeruginosa* CF2004 and after a further 24 h (aerobic, +10% CO_2_) growth inhibition determined visually. Interestingly the lowest H_2_O_2_ producer in the biofilm model—*S*. *mitis*, strongly inhibited growth of *P*. *aeruginosa* CF2004 (Fig. 4A and 4B). H_2_O_2_ mediated antagonism of *P*. *aeruginosa* CF2004 growth was also observed for *S*. *sanguinis* and *S*. *gordonii*, but was clearly weaker than that observed for the other two species. Similar results were obtained for both streptococcal colonising incubation conditions (aerobic +10% CO_2_ and anaerobic atmospheres). H_2_O_2_ was confirmed as the inhibiting substance through incorporation of catalase into the inoculum of the pioneer coloniser and the use of the *spxB* mutant (Fig. 4A, 4B, 4C and 4D).

### *S*. *oralis* is again the dominant partner when challenged with other commonly isolated phenotypes associated with CF respiratory infection

**Fig 5:**
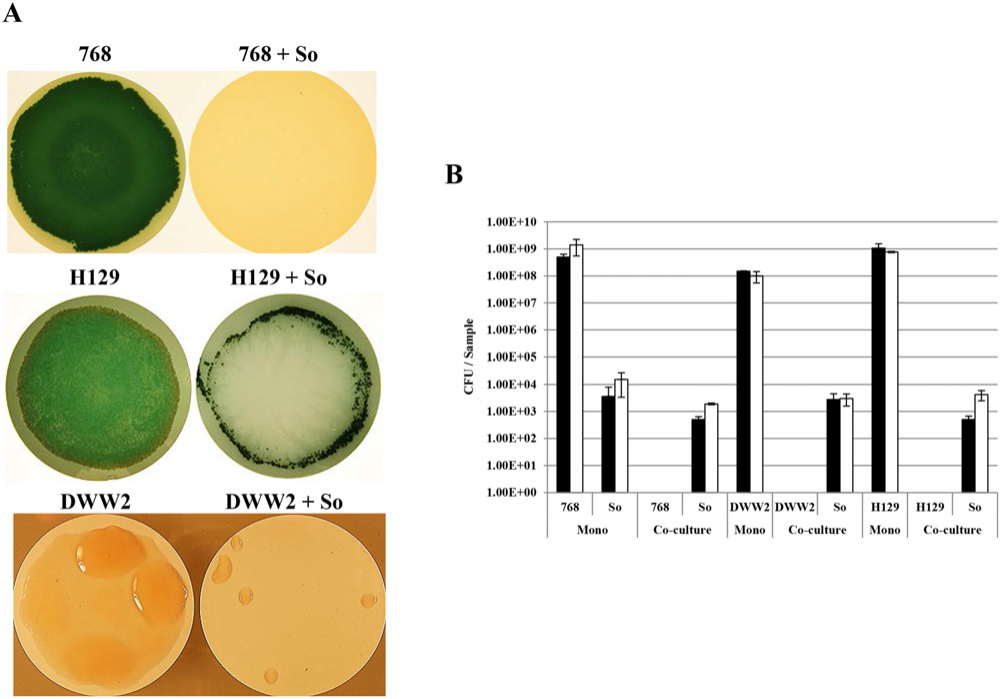
*S*. *oralis* also inhibits growth of other common *P*. *aeruginosa* CF phenotypes. Bacterial strains—768, *P*. *aeruginosa* 768; H129, *P*. *aeruginosa* H129; DWW2, *P*. *aeruginosa* DWW2; So, *S*. *oralis* ATCC 35037^T^. (A) Biofilms formed on nitrocellulose filters after 48 h (37°C, 10% CO_2_). (B) Quantitative bacteriology (CFU / filter) of samples taken from central regions of monoculture and co-culture biofilms after 24 and 48 h at 37°C (+10% CO_2_); shaded bars 24 h population, unshaded bars 48 h population. Detection limit; 1.0x10^3^ CFU/ Sample. Standard deviation shown for duplicate or triplicate samples.

Three other variants of *P*. *aeruginosa* (768, H129 and DWW2) with different phenotypes to CF2004 were challenged with *S*. *oralis* in co-culture (aerobic +10% CO_2_); DWW2, which is a mucoid CF isolate and LES variants 768 (highly pigmented due to overproduction of pyocyanin) and H129 (intermediate pyocyanin producer) [15,22]. All *P*. *aeruginosa* variants were able to form confluent biofilms when grown alone but colonies were either absent (768) or observed only at the perimeter of the filter (H129, DWW2) when cultured together with *S*. *oralis* (Fig. 5A). Again *S*. *oralis* colonies just visible to the naked eye covered the majority of the surface of the filter and their dominance (5.0 x 10^2^–4.2 x 10^3^ per sample) and absence of *P*. *aeruginosa* (below detection limit of 1 x 10^3^ per sample) in these regions was confirmed through quantitative bacteriology (Fig. 5B).

### Cooperation is observed between *P*. *aeruginosa* CF2004 and *S*. *oralis* ATCC 35037 grown together under aerobic conditions

**Fig 6:**
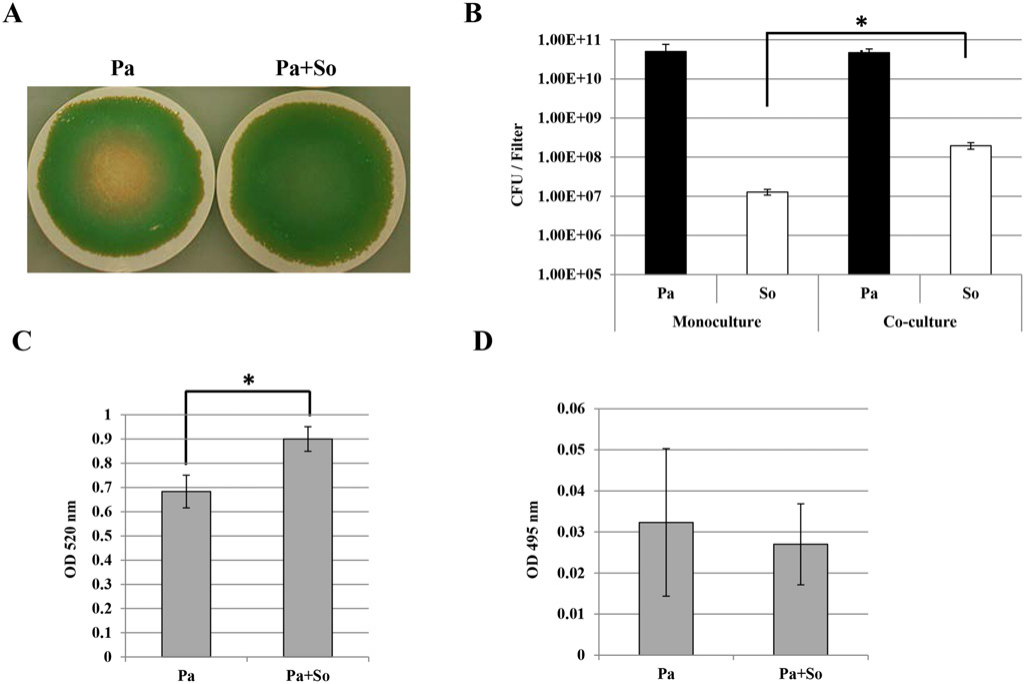
Enhancement of *P*. *aeruginosa* CF2004 virulence factor by *S*. *oralis* in biofilms incubated without added CO_2_. Bacterial strains—Pa, *P*. *aeruginosa* CF2004; So, *S*. *oralis* ATCC 35037^T^. Biofilms were grown aerobically on nitrocellulose filters for 48 h at 37°C. A) photographs and (B) quantitative bacteriology (CFU / filter) of monoculture and co-culture biofilms; shaded bars *P*. *aeruginosa* CF2004 population, unshaded bars streptococcal population. (C) Pyocyanin and (D) elastase activity in biofilms. Standard deviation of triplicate or quadruplicate cultures shown. Results significantly different from control using the two-tailed t-test are denoted with asterisks (*p<0.05, ** *p*<0.01).

As *S*. *oralis* grew poorly in air compared to atmospheres containing CO_2_ and produced significantly less H_2_O_2_, we postulated that it could form a cooperative partnership with *P*. *aeruginosa* in this environment. Mono and co-culture biofilms were grown at 37°C in air. In combination *S*. *oralis* and CF2004 formed a highly pigmented confluent biofilm (Fig. 6A), a striking contrast to the patchy biofilm this combination formed in the presence of 10% CO_2_ (Fig. 1A). As demonstrated with the other species of streptococci grown in combination in a 10% CO_2_ atmosphere, we observed comparable *P*. *aeruginosa* populations in CF2004 monoculture / co-culture biofilms (4.7–5.0x10^10^ CFU / filter), high *S*. *oralis* cell density in co-culture (2.0x10^8^ CFU / filter, 15 fold greater than in monoculture) and significantly enhanced pyocyanin levels in co-culture (Fig. 6B, *p*<0.01; 6C, *p*<0.05).

## Discussion

Previously we observed that the AGS could enhance production of elastase and pyocyanin, virulence factors associated with CF lung pathogenesis and thus disease progression when in co-culture with the LES variants CF2004 (low pyocyanin producer variant) and H129 (intermediate pyocyanin / high elastase producer) [15]. Here we show that four other streptococcal species (*S*. *oralis*, *S*. *mitis*, *S*. *gordonii* and *S*. *sanguinis*) can also survive in co-culture biofilm with *P*. *aeruginosa* CF2004 and the observed stimulation of virulence factor production show that the AGS are not the only streptococci able to potentiate the pathogenicity of *P*. *aeruginosa* CF isolates. Interestingly, contrasting observations were made for *S*. *oralis* when incubated in co-culture under two different environmental conditions; in the presence of added CO_2_ it is a lethal competitor producing enough H_2_O_2_ to reshape the dynamics of the partnership whilst in air it is a cooperator coexisting at high cell density and stimulating *P*. *aeruginosa* virulence factor production. Together, these data show that the nature of these interactions can be driven by the local environment. CF airways provide a highly variable habitat with diverse predisposing micro-environmental conditions and regional differences in microflora [23,24,25]. Whilst it is now well known that the thick dehydrated mucus that coats the CF airway has hypoxic regions, and as alveolar hypoventilation and severe hypercapnia are frequently observed in critically ill adult CF patients, it is highly likely that CO_2_ levels are also variable and in excess of that occurring in the healthy lung [23,26,27]. Therefore *S*. *oralis* has a capacity to influence CF community dynamics, both through the facilitation of lung damage by *P*. *aeruginosa* or alternatively by prevention of its colonization, that is environmentally dependent.

H_2_O_2_ is known to shape the colonization process in other ecosystems. For instance, oral commensal streptococci can produce competitive amounts of H_2_O_2_ that are involved in immigration selection during oral biofilm development and species composition has a direct impact on disease progression [28]. Similarly, interspecies interference by *Streptococcus pneumoniae* towards *Staphylococcus aureus* has been shown to be H_2_O_2_ mediated [29]. Data generated from H_2_O_2_ quantification in streptococcal monoculture biofilms (aerobic, + CO_2_) support our co-culture biofilm observations, showing *S*. *oralis* to be the highest producer of the four species examined (Fig. 3B). The lower levels of H_2_O_2_ produced by *S*. *gordonii* and *S*. *sanguinis* (Fig. 3B) could be due to repression in the biofilm mode of growth, as previously observed [30]. Interestingly, we also showed H_2_O_2_ to reduce biofilm formation in *S*. *oralis* monoculture biofilms, as a significantly higher cell density was obtained for the *spxB* deletion mutant and catalase protected biofilms. Thus this is compelling evidence that H_2_O_2_ production is also a mechanism for self-limitation of biofilm development and/or for the release of matrix components such as eDNA as observed previously for other Mitis group streptococci [28,31]. In addition we observed that *S*. *oralis* grows poorly in monoculture in air (Fig. 3A), but survives at high cell density in co-culture under this condition (Fig. 6B). There could be a number of interrelated reasons for increased *S*. *oralis* growth, including; the increased surface area created by the *P*. *aeruginosa* biofilm matrix, mutually beneficial metabolic interactions between co-colonisers and *P*. *aeruginosa* mediated detoxification of H_2_O_2_. In support of the latter, we observed that the *S*. *oralis* population can be enhanced in catalase protected co-culture biofilms when compared with unprotected controls, although a different incubation environment (aerobically +10% CO_2_) was used in that analysis (Fig. 2B). In addition the biofilm populations for the *S*. *oralis spxB* mutant are similar in mono and co-culture (Fig. 1B). Survival of *P*. *aeruginosa* in air co-cultures can be attributed to neutralization of any H_2_O_2_ present by catalase produced during its more rapid growth and by its numerically dominant population.

The observation that antagonism towards growth of *P*. *aeruginosa* is only visible after *S*. *mitis*, and to a lesser extent *S*. *gordonii* and *S*. *sanguinis*, are grown as pioneer colonisers shows that the inoculation sequence and the relative populations of these competing microbes has a major influence on community composition. Our laboratory observations showing antagonism by all four streptococcal species may presage the situation *in-vivo* in the CF lung when considered from a temporal perspective; it has been shown that streptococci of the Mitis species group that includes the species studied here, are one of the predominant groups of bacteria in the respiratory microbiome of infant CF patients, being detectable in relatively high numbers in the first month of life [32]. This mirrors the colonization pattern of the oropharynx in healthy neonates studied by Könönen and colleagues where the same group of streptococci were detectable at the earliest time point sampled after birth at 2 months [33]. Therefore, relatively early establishment of these commensal species in the oral cavity and CF lung may well be a significant factor in dictating community member acquisition, establishment and the subsequent interactions as the CF lung microbiome develops thereby delaying or promoting disease progression.

It is now accepted that the airway of a CF patient is a highly diverse and dynamic environment populated by a complex infecting microflora. It is also acknowledged that an understanding of these microbial communities, their dynamics and effects on the physiology of the infected CF lung will not only help us to identify members associated with a less pathogenic state but also to optimise therapeutic strategies for this disease [25]. The results of this study provide a window on the complexity of potential interactions between some members of the CF microbiota and the interpecies, environmental, spatial and temporal factors influencing them. Given the heterogeneity of sites within a single CF lung and between patients both in terms of environmental factors and microbial composition [24,34] it is clear that a deeper knowledge of the nature of interactive behaviour within the communities is required if we are to understand CF microbial community dynamics and evolution associated with health and disease progression.

## Supporting Information

S1 Fig H_2_O_2_ production per million streptococcal mono-culture biofilm CFU. Values displayed calculated from data presented in Fig. 3A and 3B).
(TIF)

S1 File Tables A, B, C, D and E. Raw data for Figs. 1, 2, 3, 5 and 6 respectively.
(XLSX)
