# Supplementary figures and images for: Environment and Colonisation Sequence Are Key Parameters Driving Cooperation and Competition between Pseudomonas aeruginosa Cystic Fibrosis Strains and Oral Commensal Streptococci
Source: PLoS One. 2015 Feb 24;10(2):e0115513. doi: 10.1371/journal.pone.0115513 (PMC4339374; doi:10.1371/journal.pone.0115513)

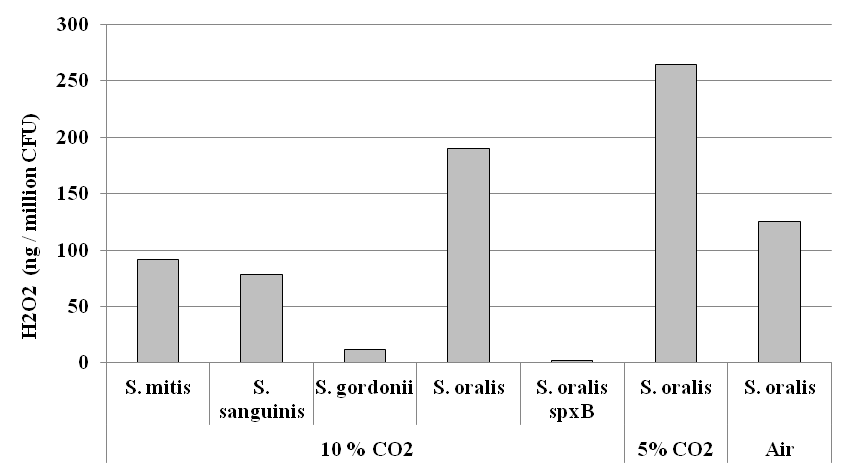

Supplement: S1 Fig — Values displayed calculated from data presented in Fig. 3A and 3B). (TIF) [file pone.0115513.s001.tif]
